# A Molecular Model for the Differential Activation of STAT3 and STAT6 by the Herpesviral Oncoprotein Tip

**DOI:** 10.1371/journal.pone.0034306

**Published:** 2012-04-03

**Authors:** Eman Dey Mazumder, Christophe Jardin, Benjamin Vogel, Elke Heck, Brigitte Scholz, Doris Lengenfelder, Heinrich Sticht, Armin Ensser

**Affiliations:** 1 Institute for Clinical and Molecular Virology, Universitätsklinikum, Friedrich Alexander University of Erlangen-Nuremberg, Erlangen, Germany; 2 Division of Bioinformatics, Institute of Biochemistry, Emil-Fischer-Zentrum, Friedrich Alexander University of Erlangen-Nuremberg, Erlangen, Germany; Hungarian Academy of Sciences, Hungary

## Abstract

Constitutive STAT signaling provides growth promoting signals in many forms of malignancy. We performed molecular modeling and molecular dynamics studies of the interaction between the regulatory Src homology 2 (SH2) domains of STAT3 and 6 with phosphorylated peptides of the herpesviral oncoprotein Tip, which facilitates Src kinase mediated STAT-activation and T cell proliferation. The studies give insight into the ligand binding specificity of the STAT SH2 domains and provide the first model for the differential activation of STAT3 or STAT6 by two distinct regions of the viral Tip protein. The biological relevance of the modeled interactions was then confirmed by activation studies using corresponding recombinant oncoproteins, and finally by respective recombinant viruses. The functional data give experimental validation of the molecular dynamics study, and provide evidence for the involvement of STAT6 in the herpesvirus induced T cell proliferation.

## Introduction

**Figure 1:**
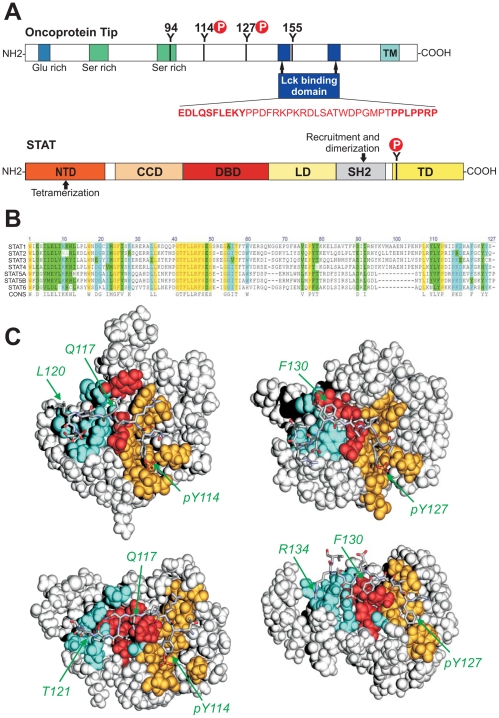
Structures of the modeled Tip-STAT complexes. (A) Tip and STAT structural domains (not to scale). (Top) From amino-terminus to carboxy-terminus of Tip the four tyrosine residues are displayed, the major phosphotyrosine residues Y114 and Y127 are also highlighted, along with the Lck binding domains; transmembrane region (TM). (Bottom) Schematic arrangement of STAT domains; N terminal domain (NTD), Coiled coil domain (CCD), DNA binding domain (DBD), Src homology 2 (SH2) domain, Linker domain (LD), Y tyrosine that stabilizes antiparallel conformation upon phosphorylation, and Transactivation domain (TD). (B) Multiple sequence alignment of human STAT SH2 domain sequences. Human STAT SH2 domains were aligned using CLUSTAL Omega [47] and displayed with Vektor NTi (version 9). The consensus is based on residues present in more than 75% of sequences; fully conserved residues are colored in yellow, those present in more than 75% in blue, homologous amino acids are colored in green (C) Structures of the modeled Tip-STAT complexes highlighting the overall complex geometry and the main interacting regions. (top left) Tip114-STAT3, (top right) Tip127-STAT3, (bottom left) Tip114-STAT6, and (bottom right) Tip127-STAT6. The STAT SH2 domain is shown in grey space-filled presentation. The three main interacting regions are defined and colored as follows: region I (binds ligand residues pY, pY+1; orange), region II (binds pY+2, pY+3; red), region III (binds pY+4 to pY+8; cyan). The Tip ligand is shown in stick presentation and key residues belonging to different interaction regions are labeled.

Signal transducers and activators of transcription (STAT) are critical signaling mediators of cytokine receptors in *Animalia*. All seven human STAT factors are predominantly activated by growth factors and cytokines, but several are also found constitutively active in cancers. The principal domain organization is identical in all the STAT members (Fig. 1A), although sequence similarities between the individual STATs are limited. The carboxyterminal part constitutes the transactivation domain (TAD), and the centre of the STAT molecule features the DNA binding domain (DBD). At the amino-terminus, a regulatory domain exists that can mediate the formation of tetramers, as in the case of STAT5 [1]; STATs are thought to exist as parallel or anti-parallel dimers that can shuttle between nucleus and cytoplasm.

The Src homology 2 (SH2) domain (Fig.1B) is arguably the most prominent domain and serves two purposes: it provides a docking domain for binding of phosphorylated tyrosine residues and it also enables homotypic interaction of STAT phosphorylated at regulatory tyrosine residues. The recognition of ligands by SH2 domains requires a phosphorylated tyrosine residue (pY) that deeply penetrates into a pocket with a high excess of basic charge. A second crucial interaction is formed by the residue pY+3, which recognizes a second pocket on the surface of the SH2 domain. In addition to these two key interactions, other residues C-terminally adjacent to the phosphotyrosine may additionally contribute to the affinity and specificity of SH2 recognition.

STAT-factors are activated by phosphorylation at the regulatory tyrosine residue, Y641 in STAT6 or Y705 in STAT3, per default by a receptor associated Janus kinase (JAK); this allows an intermolecular interaction with the SH2 domain of the second STAT subunit, thereby stabilizing the parallel STAT configuration. The enhanced interaction with DNA as well as decreased nuclear export of phosphorylated STAT lead to accumulation in the nucleus (previously described as dimerization dependent nuclear localization or translocation) [2]–[4]. In the nucleus, the phosphorylated dimers bind to the DNA targets and regulate the transcription of target genes.

STAT6 is primarily activated in response to the cytokines IL-4 and IL-13, while STAT3 mediates response to the cytokine IL-6 and IL-10. When the cytokines bind to their cognate receptors on the cell surface, the associated Janus kinases (JAK) is activated and phosphorylates conserved cytoplasmic tyrosine residues of the receptor. STAT6 docks on the phosphorylated receptor via a SH2 domain and is phosphorylated by JAKs on a conserved tyrosine residue (Tyr641 in human STAT6). STAT6 is known to play a role in several T cell responses including the development of T-helper type 2 (Th2) cells and IL-4 stimulated proliferative responses. Immunoglobulin class switching to IgE and IgG1 is promoted by STAT6 [5] along with the expression of some cell surface molecules responsible for antigen presentation by B cells. STAT6 is also functional in macrophages and dendritic cells, providing a balance of inflammatory and allergic responses. Aberrant signaling leads to constitutive activation of STAT6; this was identified in patient samples from prostate cancer tissues, Hodgkin lymphomas, adult T cell leukemia/lymphomas [6], primary mediastinal large B cell lymphomas, cutaneous T cell lymphomas.

*Herpesvirus saimiri* (HVS) is the prototypic γ2-herpesvirus and related to KSHV. HVS was isolated from the squirrel monkey (*Saimiri sciureus*) and presumably persists in the T lymphocytes of its natural host [7]. While no symptoms were described in the squirrel monkeys, other susceptible New World monkey species like common marmosets (*Callithrix jacchus*) and cottontop tamarins (*Saguinus oedipus*) develop rapidly growing T cell malignancies after experimental infection [8]. HVS strains are classified into three subgroups (A, B, C) according to sequence divergence, especially at the left end of the genome and to the transforming potential [9]. While subgroup B strains are considered the least pathogenic [10], subgroup C strains like C488 are most oncogenic and are also able to transform human T cells to antigen-independent growth *in vitro* [11]. This cell system supports a tight latency of the virus with only few viral genes being abundantly expressed [12], [13]. Among these are two oncogenes *stpC* (Saimiri transformation-associated protein C) and *tip* (tyrosine-kinase interacting protein), which are encoded by a bicistronic transcript, and four out of five genes coding for U-RNAs, that are also located near the left end of the coding region [14]–[16]. Activation of STAT3 has been reported in primate T cells transformed with the more pathogenic strains of HVS subgroups A and C [10] **,** in human T cells transformed by HVS subgroup C and also by recombinant HVS expressing the Tio oncoprotein of Herpesvirus ateles [17], [18]. In analogy to human leukemia [19], STAT3 was thus considered as one of the relevant pathways required for viral transformation and pathogenesis. In HVS of subgroup A, the StpA oncoproteins mediates STAT3 activation [20], while Tip is responsible in HVS of subgroup C [21]–[23].

Tip is a 40 kDa phosphoprotein that co-precipitated with the lymphocyte specific non-receptor tyrosine kinase Lck [24]. In order from amino- to carboxy-terminus, the Tip protein consists of a glutamate–rich region, one or two serine-rich regions, Lck binding elements commonly referred to as ligand binding domains 1 and 2 (LBD1 and LBD2) and a hydrophobic membrane anchor (Fig. 1A). A series of 3 or 4 tyrosine residues occurs in the central region of all HVS subgroup C Tip proteins, and three tyrosine residues, Y114, Y127 and Y155 in C488, are conserved [25]. Lck binds to Tip via both LBD1 (a proline-rich SH3 domain binding sequence, SH3B) and LBD2 (nine amino acids with homology to the C-terminal regulatory regions of various Src kinases, CSKH) [26]. Y127 can interact with the SH2 domain of Lck, but is not required for interaction [27], whereas both LBD1 and LBD2 are required for full Lck binding of Tip. The Tip-Lck interaction is essential for transformation by HVS [28]; it is also a prerequisite for STAT3 activation [21], [22], [29].

In recent years it has become clear that STAT factors are key signaling molecules in cell differentiation and cell growth and survival. Epigenetic processes are also involved in stabilizing and regulating these signals. In two previous studies we investigated the role of Tip Y114 mediating STAT3 activation, and the major role played by the tyrosine phosphorylation site Tip Y127 in transformed human T cells [28], [30]. To our surprise, we found that STAT3 activation is not necessary for transformation [30], as Tip Y114 mutation to phenylalanine (TipY114F) abolished the constitutive STAT3 activation observed in HVS-wildtype transformed T cells, without any negative effects on transformation. Interestingly, mutation of the major tyrosine phosphorylation site of Tip, Y127, was compatible with viral transformation, but only when IL-2 was supplemented [28]. A recent study described that Tip, when cotransfected with STAT6 into Jurkat T cells, can also interact with STAT6, relocalize and activate STAT6, and it is Tip Y127 that was required for this activation [31].

This work focuses on the interaction between Tip, STAT3 or STAT6 and Lck, which results in STAT factor activation. It is not completely understood how the herpesviral oncoprotein Tip, in complex with Lck structurally interacts with the STAT proteins, nor how this interaction facilitates the activation of the respective factor. In this study we generated a computational model using molecular dynamics simulations of individual Tip phosphotyrosine peptides in complex with STAT3 and STAT6, which were shown to mediate the Lck dependent activation. The modeling and computational studies are experimentally supported by transfection studies and by respective recombinant herpesviruses; the results implicate activation of STAT6, in contrast to STAT3, in viral growth transformation.

## Results and Discussion

### Differential Role of the Tyrosines 114 and 127 in STAT3 Recognition

**Figure 2:**
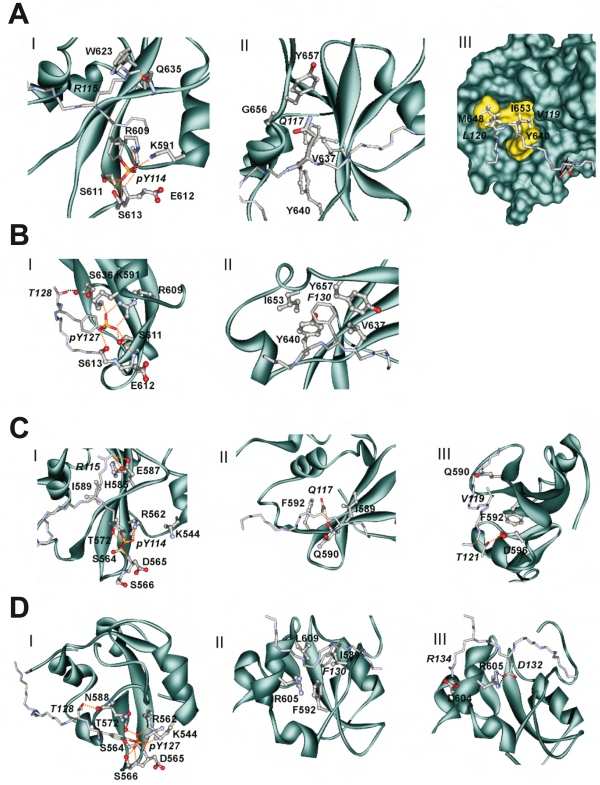
Details of the Tip-STAT interaction in the four investigated complexes. (A) Tip114-STAT3, (B) Tip127-STAT3, (C) Tip114-STAT6, and (D) Tip127-STAT6. For each complex, three panels are shown that highlight the interactions formed in region I, II, and III. The backbone topology of STAT is shown as cyan ribbon and the interacting residues are shown in ball-and-stick presentation. Tip those residues that bind to STAT are shown in stick presentation and labeled in italics. Polar interactions (hydrogen bonds, salt-bridges) are indicated as dotted red lines.

Previous experiments have indicated a role of the Tip phosphotyrosines 114 and 127 for STAT3 and STAT6 activation, although the exact role of the individual residues still needs to be clarified [28], [30], [31]. We have therefore used molecular modeling and molecular dynamics to get further insight into the SH2 binding preferences of Tip peptides comprising either a phosphorylated Y114 or Y127. The representative complex structures and the interactions deduced form the molecular dynamics simulations are shown in Fig. 2 and Table 1, respectively.

**Table 1:** Tip-Stat interactions detected in the molecular dynamics simulations.

Region	I		II		III				
**TIP114-**	**pY114**	**R115**	**P116**	**Q117**	**N118**	**V119**	**L120**	**T121**	**N122**
**STAT3**	K591 s-s	W623 s-s		V637 s-s		Y640 s-s	M648 s-s		
	R609 s-s	Q635 hs-s		Y640 m-s		I653 s-s			
	S611 s-s			Y657 hs-s					
	E612 s-m								
	S613 s-s								
**TIP114-**	**pY114**	**R115**	**P116**	**Q117**	**N118**	**V119**	**L120**	**T121**	**N122**
**STAT6**	R562 s-s	E587 s-s		L609 s-s		Q590 s-hs		D596 s-s	
	S564 s-s	I589 s-hs		I589 s-s		F592 s-s			
	D565 s-m	H585 s-s		F592 s-s					
	S566 s-s								
	T572 s-s								
**TIP127-**	**pY127**	**T128**	**T129**	**F130**	**E131**	**D132**	**A133**	**R134**	**V135**
**STAT3**	K591 s-s	S636 s-m		Y640 s-s					
	R609 s-s			Y657 s-s					
	S611 s-s								
	S613 s-s								
**TIP127-**	**pY127**	**T128**	**T129**	**F130**	**E131**	**D132**	**A133**	**R134**	**V135**
**STAT6**	K544 s-s	N588 s-m		I589 s-s		R605 s-s		D604 s-s	D608 m-s
	R562 s-s			F592 s-s					
	D565 s-m			R605 hs-s					
	S566 s-s			L609 s-s					
	T572 s-s								

Only those interactions that were stable over more than 30% of the simulation time are reported. Residues of Tip are shown in bold and their interacting partners from Stat3/6 are listed in the column below. The letters ‘m’ and ‘s’ denote whether an interaction is formed by the main-chain or side-chain atoms, respectively (e.g. m-s denotes an interaction between the main-chain of the first residue (from Tip) and the side chain of the second residue (from STAT)). ‘hs’ denotes the special situation, in which only the hydrophobic atoms but not the polar part of a sidechain is involved in an interaction. In the top line, the three main interacting regions (I to III) are indicated. Region I comprises the phosphotyrosine itself and the adjacent residue pY+1. Region II comprises residues pY+2 and pY+3. The remaining C-terminal residues (pY+4 to pY+8) constitute region III.

For two purposes, analysis started with the STAT3-Tip114 complex: Firstly, the respective interaction is known from previous experimental studies [21], [22], [29] and its physiological relevance has been demonstrated [30]. Secondly, the pY114-R-P-Q stretch conforms the Y-x-P-Q consensus sequence for high affinity STAT3 ligands [32], thus providing a reference to evaluate the outcome of the modeling study.

Consistent with the experimental data above, the STAT3-Tip114 complex forms numerous tight contacts via the *pY114-Q117* sequence stretch. The phosphotyrosine tightly interacts with two basic and additional polar residues in the binding pocket and *R115* at the pY+1 position additionally forms a π-π stacking interaction with W623 of STAT3 (Table 1, Fig. 2A). *Q117* at the pY+3 position tightly interacts with two tyrosines (Y640, Y657) of STAT3. These interactions are also stable over time and only minor fluctuations of the contacts are observed (Fig. S1).

Interestingly, additional hydrophobic interactions are formed by *V119* and *L120* at the pY+5 and pY+6 position of Tip114. These residues recognize a hydrophobic surface patch at the STAT3 surface that is formed by Y640, M648, I653, and M655 (Fig. 2A, 3A). This finding is in line with previous models of STAT3 complexed with phosphopeptide inhibitors, which also suggested that a hydrophobic residue at position pY+5 (a phenylalanine in the STAT3 dimer, and a valine in a phosphopeptide inhibitor of STAT3) can be accommodated in a hydrophobic pocket on the STAT3 surface [33]. Based on the finding that C-terminal ligand residues downstream of position pY+3 are also involved in binding, we dissected the Tip-STAT interface into three different interaction regions to facilitate the subsequent comparison of different complexes: Region I comprises the phosphotyrosine itself and the adjacent residue pY+1. Region II comprises residues pY+2 and pY+3. The remaining C-terminal residues (pY+4 to pY+8) constitute region III (Fig. 1C, Table 1). In summary, Tip114 exhibits tight contacts in all these three regions classifying it as a strong STAT3 binder.

**Figure 3:**
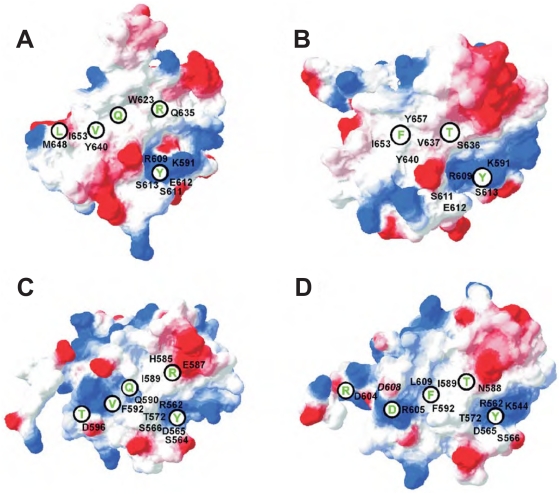
Electrostatic surface of the Stat SH2 domains and interaction sites with the Tip ligand. (A) Tip114-STAT3, (B) Tip127-STAT3, (C) Tip114-STAT6, and (D) Tip127-STAT6. Positively and negatively charged regions of the STAT surface are colored in blue and red, respectively, and the residues that interact with Tip are labeled in black. The position of the bound residues from Tip (labeled in green) is indicated by a black circle.

The high degree of complementarity between Tip114 and STAT3 becomes even more striking by comparing it to the Tip127-STAT3 complex. In STAT3, the surface patch of region III is largely hydrophobic (Fig. 3B). While this property is favorable for Tip114 binding, the polar C-terminal residues of Tip127 cannot interact with STAT3 (Table 1). In addition to the lack of interactions in region III, modeling indicates that the hydrophobic pocket appears to be too small to accommodate the bulky *F130* (Fig. 2B). This property can be seen from the large conformational rearrangement of the phenylalanine in the STAT3 pocket, which occurs in the second half of the simulation (Fig. S1). Based on these modeling studies, one would expect that only Y114 but not Y127 can form a stable interaction with STAT3.

**Figure 4:**
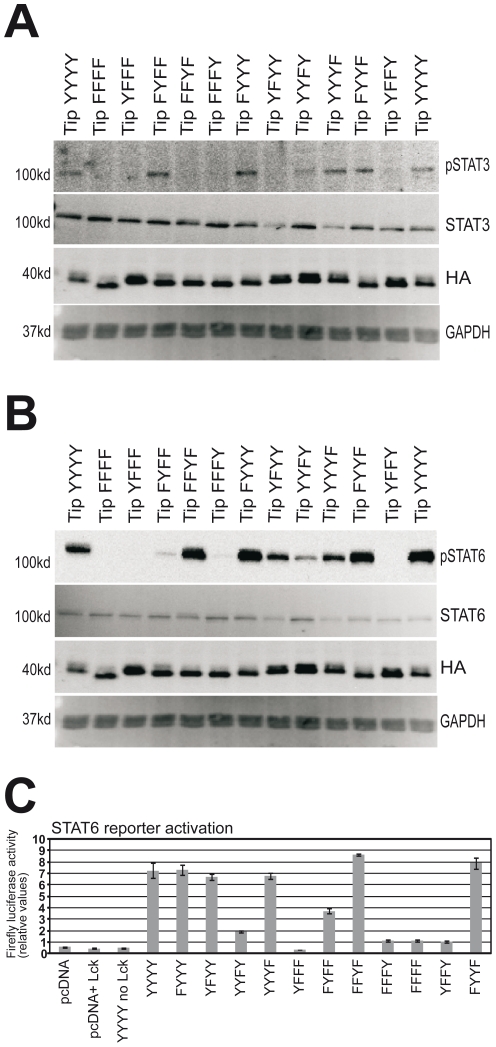
STAT3 and STAT6 factors are activated in Tip and Lck transfected cells, and the transcriptional activity STAT6 mediated by Tip. Active STAT, phosphorylated at the respective tyrosine residue required for dimerization and activation, was detected by antibodies recognizing only the phosphorylated domain. (A) STAT3 phosphorylated at Y705; pSTAT6, Tip (HA) and GAPDH were detected by multicolor fluorescent immunoblot without stripping of the membrane; STAT3 was detected in a parallel experiment using the same cell protein lysate (B) STAT6 phosphorylated at Y641. STAT6, pSTAT6, Tip(HA) and GAPDH were detected by multicolor fluorescent immunoblot without stripping of the membrane. A representative example of several independent immunoblotting experiments is shown. (C) For each experiment dataset (triplicate values) firefly luciferase activity was normalized to the activity of renilla luciferase transfection control, the mean then mean and SD values were calculated and are shown for a representative experiment.

In line with structural analysis study, we wanted to re-affirm the prominent role played by the Tip residue Y114 to stimulate the transcription factor STAT3. 293T cells were transfected with STAT3 along with Lck and Tip expression constructs including the wildtype Tip variant and a range of mutant variants where the four tyrosine residues were replaced with phenylalanine in different combinations. Immunoblot analysis data with phospho-STAT3 antibody revealed that only the presence of Tip residue Y114 can drive STAT3 phosphorylation (Fig. 4A). This first set of experiment on STAT3 activation status under the influence of Tip-Y114 is in line with our previous observation that that this residue is solely responsible for STAT3 transcriptional activation in HVS-transformed human T cells [30]. Mutation of Tip residue Y127 did not have an effect on the activation of STAT3, underlining the validity of our structural and modeling analysis.

### Structural Role of Tip Tyrosine Residues Y114 and Y127 in the Recognition of STAT6

In the next simulation, we analyzed whether Tip114 can recognize STAT6 in an analogous fashion to STAT3. The interactions formed in the phosphotyrosine pocket are similar in both complexes (Table 1, Fig. 2A,C). The adjacent *R115* (pY+1) also forms tight interactions in the STAT6 complex including side chain stacking with H585 and a salt-bridge with E587. *Q117* (pY+3) of Tip114 is located in a hydrophobic pocket of the STAT6 surface (Fig. 2C). The residues downstream of *Q117* also form interactions with STAT6, although the nature of the interaction is clearly distinct from the Tip114-STAT3 complex (Table 1; region III). These differences can be explained by the fact that the hydrophobic surface patch present in STAT3 is replaced by charged residues (R600, R605, D608) in STAT6 (Fig. 3A,C). Consequently, the hydrophobic C-terminus of Tip114 adopts an alternative conformation to allow for an interaction with Q590 and F592 of STAT6. These different surface properties of STAT3 and STAT6 and the major contact points with Tip114 are compared in Fig. 2. The STAT6 surface is more polar and therefore the number of hydrophobic contacts formed by *V119* is drastically reduced, compared to the Tip114-STAT3 complex. *L120* does not participate in STAT6 binding, while the polar *T121* at the pY+7 position can form a hydrogen bond with D596 (Table 1; Fig. 2C). Thus, modeling supports that Tip114 is also capable to interact with STAT6, although the lower number of contacts in region III suggests an overall weaker interaction compared to the Tip114-STAT3 complex.

We also investigated the structural basis for an interaction of STAT6 with Tip127. In addition to the contacts formed by the phosphotyrosine itself, *F130* at the pY+3 position tightly packs into a pocket that is mainly formed by the hydrophobic residues I589, F592, and L609 (Fig. 2D). The nonpolar part of the R605 sidechain is also involved in this interaction, while the polar sidechain of R605 interacts with *D132* at the pY+5 position. A second salt-bridge in region III is formed between D604 and *R134* at the pY+7 position (Fig. 2D). Thus, the complementarity of the interacting surface patches is higher for Tip127-STAT6 compared to Tip114-STAT6 (Fig. 3C,D). While Tip127-STAT6 can form two salt-bridges in region III, Tip114 can only form hydrophobic *V119*-F592 contacts as well as a *T121*-D596 hydrogen bond. The findings, together with the tighter interaction of *F130,* compared to *Q117* at the pY+3 position, suggests that phosphorylated Tip127 represents the main STAT6 binding site.

We wanted to validate this prediction on the role of pY114 and pY127 biochemically, which led us to conduct transfection of 293T cells with STAT6, Lck and Tip along with its mutant variants. The mutant Tip-FYFF (only Tip-Y114 residue) and Tip-FFYF (only Tip-Y127 residue) individually had the ability to restore STAT6 phosphorylation, although the degree of restoration varied. Tip-Y127 by itself was shown to have a stronger capacity to phosphorylate STAT6 than Tip-Y114 (Figure 4B). The replacement of Tip residues Y114 and Y127 by phenylalanine (YFYY and YYFY respectively) lessened the degree of STAT6 phosphorylation. A double mutant of the Tip construct, in which both the tyrosine residues Y114 and Y127 were replaced with phenylalanine (Tip-YFFY), almost completely rendered the STAT6 inert. Taken together, these studies confirm a role for both Tip-Y114 and Tip-Y127 in phosphorylating STAT6, but in line with the modeling predictions Tip-Y127 plays the more significant role.

### Tip-Y127 Driven STAT6 Activation

Following STAT6 phosphorylation experiments, we interrogated the transcriptional activity of STAT6-specific luciferase reporter under the influence of Tip (Fig. 4C). 293T cells were transfected with STAT6-responsive luciferase reporter plasmid together with Lck and wildtype Tip along with its previously used mutant variants. As shown in the figure, wildtype Tip (YYYY) could strongly enhance STAT6 transcriptional activity in vitro. As long as both residues Y114 and Y127 were retained in the Tip expression construct, we found an induction of STAT6 to be consistent and similar to its wildtype variant. A threefold reduction in STAT6 activity was seen when tyrosine residue Tip Y127 is phenylalanine replaced (Tip-YYFY) indicating the role of Y127 for activation. This finding is further substantiated by the detected multifold increase in induction of STAT6 activity observed for a Tip expression construct just containing a single tyrosine residue at position 127 (Tip-FFYF). A dominant effect of Tip-Y127 on STAT6 activation was revealed from the above experiment; with Tip-Y114 also shown to play a role of a lesser degree in the induction process of STAT6 *in vitro*.

### Lck is Required for STAT6 Activation

**Figure 5:**
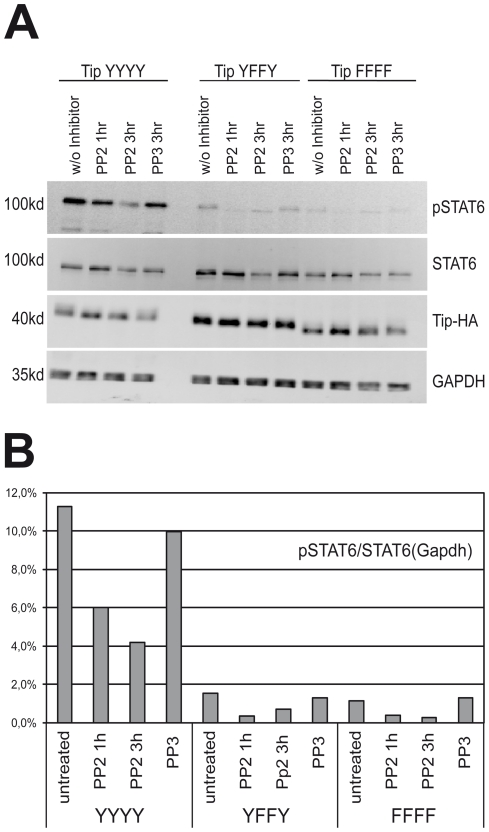
Specific inhibition of Src-Tyrosine kinase reduces STAT6 activation. (A) Tip, Lck and STAT6 transfected 293T cells were treated with PP2 or the non-active control substance PP3 (10 µM in DMSO each). STAT6, pSTAT6, Tip and GAPDH were detected by fluorescent immunoblotting on the same membrane. (B) The ratio of pSTAT6 to total STAT6 was normalized to the GAPDH signal. The ratio of STAT6-pY641 (clone 18, detected with anti mouse Dylight-647), total STAT6 (polyclonal rb-anti-STAT6 (M-20) with anti-rabbit-Dylight-488) was calculated and normalized to the GAPDH loading control (biotinylated goat anti-GAPDH/Strepavidin-Alexa-555).

Tip directly binds to the Src family tyrosine kinase Lck, which is a key regulator for T cell activation [24], [28]. In order to establish that STAT6 activation via oncoprotein Tip is mediated by the Src family kinase Lck, we went further to explore the effect of Src kinase inhibitors PP2 on STAT6 activation. 293T cells were co-transfected with STAT6, Lck and Tip expression constructs which includes its wildtype along with a subset of mutants used for the previous experiments. Post harvesting, the cells were treated with PP2 inhibitors or PP3 control at indicated time points before they were harvested for further experiments. After initial trials, we aimed to look for a comparison between the inhibitory effects posed after 1 hr and 3 hr time points, as longer exposure substantially altered total cellular viability and protein expression. Quantitative immunoblot data with the PP2/PP3 treated protein lysates at different time points (Fig. 5) clearly showed that STAT6 phosphorylation is indeed affected after 1 and 3 hrs under the influence of wild type Tip variant (Tip-YYYY). The mutants Tip-YFFY (Y114F/Y127F) and Tip-FFFF served as a negative control, where most of STAT6 activation was abrogated as expected by the mutation alone; interestingly, a PP2 sensitive baseline activity remained detectable. This could indicate that the interaction of Tip with STAT6 is not solely dependent on phosphotyrosine interaction, because Tip forms very extensive contacts at positions Y+3 to Y+8 in the Tip127-peptide (Fig. 1C, 2, 3).

### Analysis of the Role of Tip Tyrosine Residues in Virus Induced T Cell Transformation Using Recombinant Viruses

**Figure 6:**
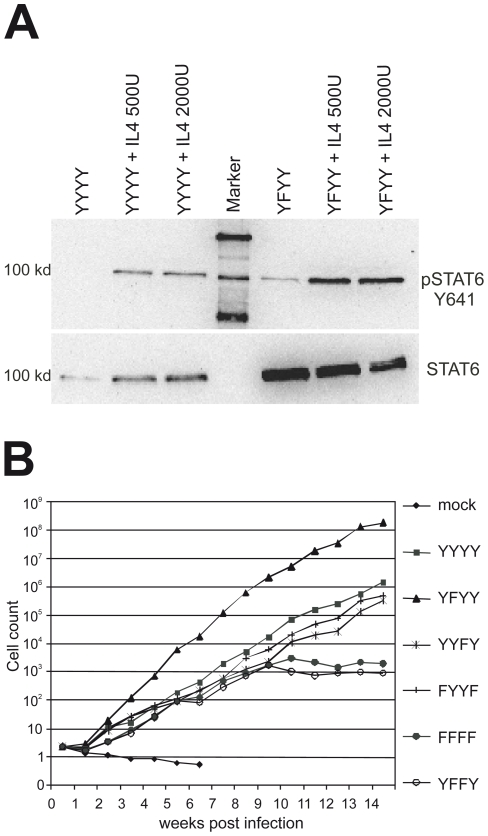
Viruses expressing Tip deficient in STAT6 activation fail to growth transform human T cells. (A) Pairs of cell lines from identical donors are shown. Values of three different HVS transformed cell lines (2 donors #1587, 1611) are shown relative to the minimal transcript level detected for each cell line. (B). Human cord blood lymphocytes were infected with recombinant HVS strain C488 viruses, and maintained in medium supplemented with Interleukin-2. Cell growth was monitored and growth curves were calculated as described previously [28]. YYYY - recombinant wild type HVS in which Tip has four tyrosine residues Y94, Y114, Y127 and Y155; YFYY - mutant Y114F (tyrosine Y114 mutated to phenylalanine F); YYFY- mutant Y127F (Y127 mutated to F); YFFY- mutant Y114F and Y127F; FYYF - mutant Y94F and Y155F; FFFF - all four tyrosines mutated.

Recombinant HVS carrying Tip proteins with tyrosine to phenylalanine (Y to F) mutations were generated by cotransfection of overlapping cosmids into OMK cells and tested for their ability to transform human T cells. Two sets of experiments with 4 donor each were performed and T cell growth was monitored; this revealed that Y114F (YFYY) lead to a more rapid initial expansion of T cells, while the additional mutation of Y127F (YFFY), or of all tyrosines (FFFF) resulted in cessation of growth after the initial phase of activation that is commonly observed in all HVS infected cells (Fig. 6).

While we can only speculate on reasons for the increased proliferation of the YFYY mutant, which is reminiscent of the growth behavior of HVS-Tio recombinant virus [17], the requirement of the two residues 114 and 127 that are able to mediate STAT6 activation hint at a relevant role of STAT6 in the transformation of human T cells. In these YFYY transformed cells we also observed a stronger expression of STAT6, as well as an increased baseline phosphorylation of STAT6 that could be further enhanced along the IL-4 signaling pathway. It would be interesting to observe the effects of STAT6 inhibition in such transformed cell lines, however, we could not knock down STAT6 by RNAi, and there are not specific inhibitors of STAT6 signaling. The Src kinase inhibitor PP2 that we used in our transfection study also abrogates other forms of Tip/Lck mediated signaling, e.g. via the T cell receptor zeta chain [34] and the general Src kinase inhibition is toxic to the HVS transformed cells and other T cell lines.

In summary, we have provided a molecular model for STAT3 and STAT6 SH2 ligand interaction of the viral oncoprotein Tip, and we support the model with biochemical, as well as functional data. The results point at a role of STAT6 activation in facilitating the growth of virus transformed T cells, and possibly other lymphoid malignancies.

## Materials and Methods

### Ethics Statement

Human cord blood was obtained from the public cord blood bank at the Section of Transfusion Medicine, Erlangen, in an anonymous manner. The donors explicitly consent that these can be used anonymously in research in case that materials are unsuitable for long term banking, e.g. due to a lack of sufficient amount or other reasons. Research on this factually anonymous material is not subject to §15 of the Medical Association’s professional code of conduct and is not requiring approval by the ethics committee in individual cases.

### Molecular Modeling of the STAT-peptide Complexes

For each transcription factor STAT3 and STAT6, two complexes were modeled with the peptide ligands named Tip114 and Tip127. The two peptides Tip114 and Tip127 correspond to the strain C488 of Tip with the phosphorylated tyrosines (pY) respectively at positions Y114 (pY114) and Y127 (pY127). Previous structural studies have shown that the residues N-terminally adjacent to the phosphotyrosine (pY) are generally not involved in SH2 domain recognition while the C-terminally adjacent residues frequently play a role in mediating binding specificity [35]. To take this observation into account, the two Tip peptides in this study were 9 amino acids long, starting with residue pY-1 and ending with residue pY+7, and had the respective amino acid sequences: *113*-G-pY-R-P-Q-N-V-L-T-*121*, and *126*-G-pY-T-T-F-E-D-A-R-*134*.

The models for the STAT3-Tip complexes were based on the crystal structure of STAT3 [PDB entry 1BG1 [36]]. This structure represents an activated STAT3 homodimer, in which the Y705-phosphorylated C-terminus is bound to the SH2 domain. For modeling, one SH2 domain (residues 584–688) with its interacting C-terminus (residues 704–712) was first extracted from the crystal structure. In the following step, the sequence of the STAT3 C-terminus was changed with Sybyl7.3 (Tripos Inc.) to match the sequence of either Tip114 or Tip127. Since there is no crystal structure yet available for STAT6, the respective SH2 domain was first modeled using the closest homologue of known 3D-structure, which is an unliganded STAT5a [PDB entry 1Y1U] [37]. The Tip-peptides were subsequently added to this model according to the geometry present in the respective Tip-STAT3 complexes. Subsequent molecular dynamics simulations of all complexes were carried out for structure refinement and to assess the conformational stability of the Tip-STAT interaction.

### Molecular Dynamics (MD) Simulations

Separate molecular dynamics simulations were performed for each STAT-peptide system with the AMBER9 package [38] and the parm99SB force field [39], [40] following an established protocol [41]. Acetyl and N-methyl groups were added at the N- and C-termini of the proteins and peptides to reduce terminal charge effects during the simulations. For the phosphorylated tyrosines, we used the parameters developed in our group [42]. All systems were neutralized with an appropriate number of counterions, and solvated with TIP3P water molecules in a box of at least 10 Å between the solute and the borders. The systems were minimized in two-steps, and heated up to 298 K in 1 ns using SHAKE constraints on hydrogens and small restraints on the Cα atoms. For each system, 24 ns of production run were carried out and coordinate snapshots were saved every 1 ps.

Salt bridges and hydrogen bonds between the SH2 domain and the peptides were identified using VMD [43]: the cut-off were 3.2 Å between oxygen and nitrogen atoms of the salt bridged residues, 3.0 Å between the heavy atoms involved in hydrogen bonding, and an angle cut-off of 120 degrees. A cutoff of 5.0 Å between the heavy atoms was used to define hydrophobic interactions. The programs RasMol [44],VMD [43], DS Viewer Pro (Version 6.0), and Povray (Version 3.6 for Windows) were used for structural analysis and visualization. The final figures were prepared with Adobe Photoshop CS and CorelDRAW 12.

### Plasmids and Recombinant Viruses

pCDNA-HA-Tip containing the open reading frame of Herpesvirus saimiri C488 tagged at the amino-terminus with HA-epitope in a modified pcDNA3 backbone has been described [28]. By applying site directed mutagenesis, respective mutants of Tip were obtained where tyrosines residues 94, 114, 127, and 155 are substituted with phenylalanine at different positions (YYYY, FFFF, YFFF, FYFF, FFYF, FFFY, FYYY, YFYY, YYFY, YYYF, FYYF, YFFY). All constructs were sequence verified. The pcDNA4-STAT6-mychis expression construct was a gift of Frank Neipel. The same mutations were introduced into a cosmid 331 encompassing the left terminal region of the HVS genome, and recombinant Herpesvirus saimiri strain C488 were generated by cotransfection of overlapping cosmids into permissive OMK cells as described [14], [28], [30].

### Cell Culture and Transfection

Adherent OMK (ATCC CRL-1556), 293T (DSMZ ACC 635) and HeLa cells (DSMZ ACC 57) were cultured using Dulbecco’s Modified Eagle Media (DMEM) supplemented with 10% fetal calf serum (FCS), antibiotics and glutamine. For transfection, HeLa and 293T cells were split 24 hours prior to transfection and grown to 70–80% confluence in 6-well plates, 10 cm tissue culture dishes, or in 4-well chamber-slides. Plasmid DNA was diluted in OptiMEM-I (Invitrogen) without antibiotics, and cationic lipid transfection reagents Fugene HD (Roche Diagnostics) or Lipofectamine 2000 (Invitrogen) were added at a ratio of 2 µl or 1 µl per µg DNA, respectively. Transfection was done with 1–3 µg total DNA per single well of a 6-well plate, 10–20 µg DNA per 10 cm dish, 1 µg per chamber slide well. The appropriate empty vector was used to equalize the amount of plasmid DNA per sample in each experiment. Treated cells were cultured in complete medium without antibiotics and harvested at the indicated time points by washing with phosphate-buffered saline (PBS) and lysis in RIPA buffer. Electroporation was carried out with a Bio-Rad gene pulser Xcell (Bio-Rad, München, Germany). Jurkat T cells (ATCC TIP-152) were transfected at 250 V, 1500 µF. Treated cells were cultured in complete medium without antibiotics and harvested at the indicated time points by washing with phosphate-buffered saline (PBS) and lysis in RIPA buffer.

### Lymphocyte Culture and Transformation

Human cord blood was obtained from the public cord blood bank at the Section of Transfusion Medicine, Erlangen, in an anonymous manner. It was tested for absence of HIV, Hepatitis B and C infection. The primary human cord blood lymphocytes (CBL) of different donors were infected and transformed with *Herpesvirus saimiri* strain C488 [11], [45] as described [28]. Human CBL were isolated by selective sedimentation of erythrocytes, and then stimulated with 1 µg/ml PHA in cell culture medium. After 24 hours, 10 units/ml exogenous IL-2 (Roche Diagnostics, Mannheim, Germany) were added and on the next day, the cells were infected with the recombinant HVS C488 or wild type, and maintained in 45% RPMI 1640 and 45% Panserin (PAN) medium supplemented with 10% fetal calf serum (PAN) and the following additives: 10 U/ml recombinant human IL-2 (Roche Diagnostics), 1 mM sodium pyruvate (PAN), 50 µM monothioglycerol (Sigma), 20 nM bathocuproine disulfonic acid (Sigma), 350 µg/ml glutamine and 100 µg/ml gentamycin. Cell culture densities were determined by automated cell counting (Beckman-Coulter Z2, Krefeld, Germany) and growth transformation was assessed microscopically and by the observation of accelerated growth over a period of at least three months after infection. Non-infected control cells that were cultivated in parallel usually ceased growing after 3–6 weeks. Growth curves were calculated as described [46]. The presence of HVS DNA in multiple viral copies in the transformed cells was verified by semi-quantitative PCR. Transformed T cell lines were also analyzed by PCR and restriction mapping to confirm the presence of the specific viral genotype, and by flow cytometry of CD3, CD4, CD8 T cell surface markers using anti-human monoclonal antibodies (Biolegend) on a FACS-Calibur flow cytometer (Becton Dickinson). FACS data analysis was performed with FCS Express 3 software (De Novo Software, Thornhill, Ontario, Canada).

### Immunoblot Analysis

Cells were washed once in cold PBS and lysed at 4 °C in RIPA buffer (50 mM HEPES, 150 mM NaCl, 1 mM EDTA, 10 mM Na_4_P_2_O_7_, 10% glycerol, 1% Triton X-100, 1 mM PMSF, 1 µg/ml Leupeptin, 10 µg/ml Aprotinin, 10 mM NaF, 1 mM Na_3_VO_4_). or in 1x TNE buffer (50 mM Tris pH 8.0, 150 mM NaCl, 1% NP-40, supplemented with 1 mM sodium orthovanadate (Na_3_VO_4_), 10 µg/ml of aprotinin, and 10 µg/ml of leupeptin (Sigma-Aldrich, Taufkirchen, Germany). Total protein concentration was determined by the bichinoninic acid assay (Pierce, Rockford, IL). Extracts corresponding to 20 or 50 µg total cellular protein were loaded on 8%, 12% or 15% SDS-polyacrylamide gels, blotted on standard or low fluorescent polyvinylidene fluoride membrane PVDF (Immobilon-P or -FL, Millipore), and blocked with 5% PBST-NFDM (phosphate-buffered saline, pH 7.4, 0.1% Tween-20, 50 g/l non-fat dry milk) or 2.5% NET-gelatine (150 mM NaCl, 5 mM EDTA, 50 mM Tris-HCl, 0.05% Triton X-100, 25 g/l gelatine) and probed with antibodies directed against HA (16B12, ms, Covance) and Myc epitopes (9E10, ms), STAT1 (ms), STAT3 (ms), STAT3-pY705 (ms) STAT6 (rb) (Santa Cruz); STAT1-pY701 (ms), STAT6-pY641 (ms) (BD Biosciences) (Cell Signaling Technology); Equal loading of the lanes was confirmed using either a biotinylated goat anti-GAPDH (Genscript), mouse anti-actin-HRP (Genscript) or mouse anti-actin antibody (Abcam). After thorough washing in PBS-Tween or NET-gelatine, immunoblots were incubated for 1 h blocking buffer with secondary reagents coupled to fluorescent dyes (goat anti-mouse DyLight-488 or -647, goat anti-rabbit DyLight-488 or -647, Thermo; streptavidin-Alexa-555, Invitrogen) or horseradish peroxidase (swine anti-mouse-HRP, swine anti-rabbit-HRP, Dako, Hamburg, Germany). Bands were visualized by enhanced chemiluminescence according to the manufacturer’s instructions (GE Healthcare) or epifluorescence on a Kodak Imagestation 4000 MM pro (Raytest, Straubenhardt, Germany).

### Luciferase Reporter Assay

The reporter plasmid contains a STAT6 consensus binding site upstream of a luciferase gene. Co-transfections included 10 µg of reporter DNA and 40 µg of expression construct. After 48 h, cells were harvested and lysed in 100 mM K_3_PO_4_ containing 0.1% Triton X-100 for 30 min at room temperature. Upon injection of 100 µl of assay buffer (200 mM Tris-HCl, 15 mM MgSO_4_, 0.1 mM EDTA, pH 8.0, 1 µM dithiothreitol, 2 µM ATP, 75 µM D-luciferin), luminescence was measured with a Microplate Luminometer (Orion II, Berthold Detection Systems, Pforzheim). For data analysis, the raw data were normalized to the protein level of the sample. Relative STAT6 activity in percent was calculated with the relative response ratio ((sample − negative control) × 100%)/(positive control − negative control)). Results are presented as the mean of multiple independent experiments ± S.E.

## Supporting Information

Figure S1 **Interactions formed by the residue at the pY+3 position (Q117 in Tip114 and F130 in Tip127) during the final 15 ns of the molecular dynamics simulation.** (A) Interactions of Q117 with V637 (black), Y640 (red), and Y657 (green) of STAT3. All contacts show only minor fluctuations indicating that Q117 forms multiple stable interactions in the respective complex. (B) Interactions of F130 with V637 (black), Y640 (red), and Y657 (green) of STAT3. For all contacts investigated, significant distance fluctuations are detected (in particular for the F130-Y640 interaction shown in red). This indicates that F130 cannot stably be accommodated in the hydrophobic binding pocket of STAT3. (C) Interactions of Q117 with F592 (black), I589 (red), L609 (green), and Q590 (blue) of STAT6. After some initial conformational changes, F130 forms multiple stable interactions in the respective complex. (D) Interactions of F130 with F592 (black), I589 (red), L609 (green), Q590 (blue), and R605 (yellow) of STAT6. All contacts show only minor fluctuations indicating that F130 forms multiple stable interactions in the respective complex.
(DOC)
