# Supplementary figures and images for: A Molecular Model for the Differential Activation of STAT3 and STAT6 by the Herpesviral Oncoprotein Tip
Source: PLoS One. 2012 Apr 3;7(4):e34306. doi: 10.1371/journal.pone.0034306 (PMC3320567; doi:10.1371/journal.pone.0034306)

# Figure S1

**A**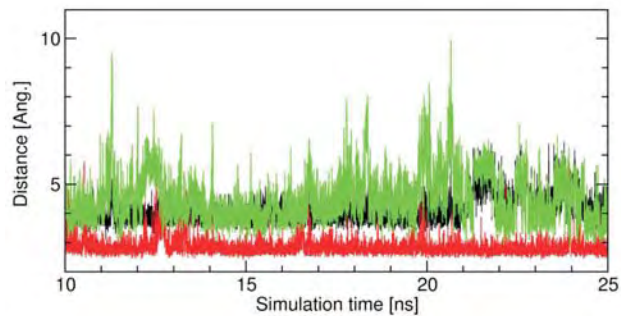**B**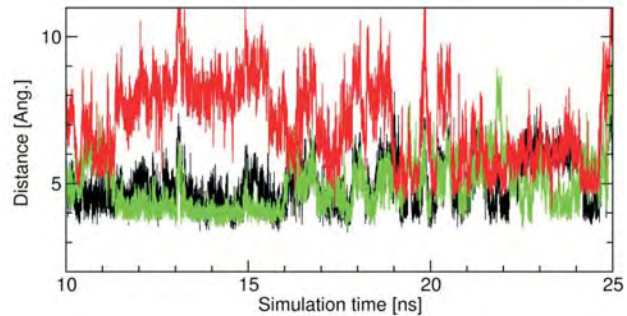**C**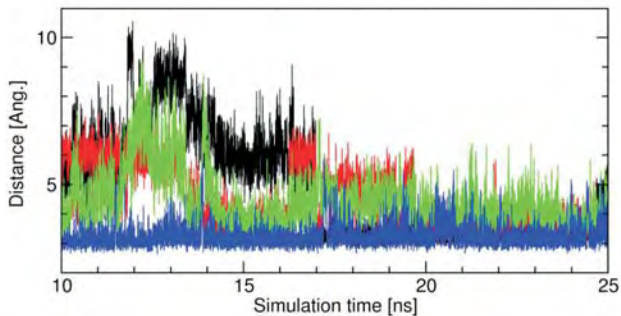**D**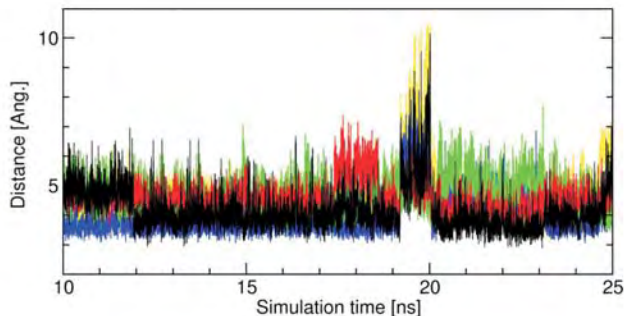

Supplement: Figure S1 — Interactions formed by the residue at the pY+3 position (Q117 in Tip114 and F130 in Tip127) during the final 15 ns of the molecular dynamics simulation. (A) Interactions of Q117 with V637 (black), Y640 (red), and Y657 (green) of STAT3. All contacts show only minor fluctuations indicating that Q117 forms multiple stable interactions in the respective complex. (B) Interactions of F130 with V637 (black), Y640 (red), and Y657 (green) of STAT3. For all contacts investigated, significant distance fluctuations are detected (in particular for the F130-Y640 interaction shown in red). This indicates that F130 cannot stably be accommodated in the hydrophobic binding pocket of STAT3. (C) Interactions of Q117 with F592 (black), I589 (red), L609 (green), and Q590 (blue) of STAT6. After some initial conformational changes, F130 forms multiple stable interactions in the respective complex. (D) Interactions of F130 with F592 (black), I589 (red), L609 (green), Q590 (blue), and R605 (yellow) of STAT6. All contacts show only minor fluctuations indicating that F130 forms multiple stable interactions in the respective complex. (DOC) [file pone.0034306.s001.doc]
